# Liver Transplantation for Alcohol-Related Liver Disease in 2026: Phenotypes, Selection, and Post-Transplant Care

**DOI:** 10.3390/jcm15207840

**Published:** 2026-10-11

**Authors:** Alberto Ferrarese, Giovanni Perricone, Laura Marta Vivian, Alberto Zanetto, Guido Viel, Patrizia Burra, Alexandre Louvet, Giacomo Germani

**Affiliations:** 1 Multivisceral Transplant Unit, Padua University Hospital, Via Giustiniani 2, 35122 Padua, Italy; 2 Department of Surgery, Oncology and Gastroenterology, Padua University, 35122 Padua, Italy; 3 Hepatology and Gastroenterology Unit, ASST Grande Ospedale Metropolitano Niguarda, 20162 Milan, Italy; 4 Gastroenterology, Padua University Hospital, 35122 Padua, Italy; 5 Legal Medicine and Toxicology, Department of Cardiac, Thoracic, Vascular Sciences and Public Health, Padua University, 35122 Padua, Italy; 6 CHU Lille, Lille University, 59037 Lille, France

**Keywords:** alcohol-related liver disease, liver transplantation, alcohol-related hepatitis, acute-on-chronic liver failure, phosphatidylethanol, return to alcohol use

## Abstract

Liver transplantation (LT) for alcohol-related liver disease (ArLD) has evolved from a pathway centered on decompensated cirrhosis and prolonged abstinence to one that also encompasses severe alcohol-related hepatitis (sAH) and alcohol-related acute-on-chronic liver failure (ACLF). This change reflects the growing burden of ArLD and evidence that carefully selected patients with acute presentations can achieve excellent post-transplant survival. The traditional six-month abstinence rule has limited value as a stand-alone predictor of harmful post-transplant alcohol use. International guidance increasingly supports individualized multidisciplinary assessment of alcohol use disorder (AUD), psychiatric comorbidity, social vulnerability, insight, and treatment engagement, although listing requirements remain heterogeneous. Abstinence nevertheless remains a disease-modifying intervention that may promote clinical improvement, hepatic recompensation, and delisting in selected candidates. After LT, long-term care should integrate prevention and treatment of harmful alcohol use with surveillance and management of graft-related, cardiometabolic, oncologic, infectious, and other medical complications. This narrative Perspective critically appraises LT for ArLD in 2026 and proposes a phenotype-informed framework integrating medical urgency, dynamic reassessment, alcohol-use biomarkers, and longitudinal transplant–addiction care.

## 1. Introduction

Alcohol-related liver disease (ArLD) is a well-established indication for liver transplantation (LT), particularly in Western countries. Historically, candidacy was framed mainly around patients with decompensated cirrhosis who had achieved a predefined period of abstinence. For those with persistent decompensation despite alcohol cessation and optimal medical management, LT provides excellent long-term survival [1].

Over the past decade, changing alcohol-consumption patterns and evidence supporting LT without prolonged pre-transplant abstinence in carefully selected patients have reshaped this pathway. US national data show a disproportionate burden of severe ArLD among adults younger than 35 years and women [2]. ArLD remains a leading indication for LT in Europe, albeit with substantial regional heterogeneity, and programs increasingly evaluate patients with sAH or alcohol-related ACLF while integrating addiction care before and after LT [3,4]. The United Kingdom reflects this broader Western pattern, while ArLD has also become more prominent in Japan, although its proportional contribution remains lower than in Western systems [5,6].

The contemporary challenge is therefore not to apply a single abstinence threshold to all candidates, but to identify those likely to derive meaningful benefit from LT, those who may improve with alcohol cessation and medical optimization, and the intensity of addiction and psychosocial care required throughout the LT continuum. This Perspective reviews transplant-relevant phenotypes and presents the authors’ proposed phenotype-informed framework for assessment and longitudinal care (Table 1). This framework is a pragmatic synthesis developed by the authors rather than an established global policy standard and should be adapted to national regulation, allocation policy, organ availability, and local resources.

### Scope and Literature Selection

This article is a narrative Perspective rather than a systematic review. We conducted targeted searches of PubMed/MEDLINE and Scopus for English-language human studies published from 1 January 2020 through 31 August 2026, supplemented by reference-list screening and searches for European Association for the Study of the Liver (EASL), American Association for the Study of Liver Diseases (AASLD), American Society of Transplantation (AST), International Liver Transplantation Society (ILTS), and national policy documents. Earlier landmark studies and guidelines were retained when needed to contextualize current practice. Search strings combined (“alcohol-related liver disease” OR “alcohol-associated liver disease” OR “alcohol-related hepatitis” OR “alcohol-associated hepatitis”) with (“liver transplantation” OR “early liver transplantation” OR “acute-on-chronic liver failure” OR “recompensation” OR “alcohol use disorder” OR “addiction treatment” OR “return to alcohol use” OR “phosphatidylethanol” OR “ethyl glucuronide” OR “post-transplant care”). We prioritized guidelines and consensus statements, systematic reviews and meta-analyses, multicenter cohorts, prospective studies, and pivotal trials; small single-center or pilot studies were retained only for emerging interventions or important evidence gaps. Non-human studies, conference abstracts without a full report, and studies without clinically interpretable outcomes were excluded. No protocol was registered, and no duplicate screening, formal risk-of-bias assessment, or quantitative pooling was undertaken.

## 2. Transplant-Relevant Phenotypes of Alcohol-Related Liver Disease

### 2.1. Decompensated Alcohol-Related Cirrhosis

Decompensated alcohol-related cirrhosis remains the most established transplant-relevant phenotype. Decompensation is defined by ascites, variceal bleeding, or overt hepatic encephalopathy and may be followed by spontaneous bacterial peritonitis or other bacterial infections, acute kidney injury or hepatorenal syndrome, and progressive nutritional and functional decline. Malnutrition, sarcopenia, and frailty are particularly common and may increase morbidity and mortality independently of conventional liver-severity scores. Portal hypertension drives the transition from compensated to decompensated cirrhosis. Continued alcohol exposure may further worsen liver injury, gut–liver axis dysfunction, and intrahepatic and systemic inflammation, thereby maintaining vascular resistance and susceptibility to decompensation. Conversely, prolonged abstinence has been associated with improved outcomes across stages of portal hypertension and, in alcohol-related cirrhosis, with a lower hepatic venous pressure gradient and reduced inflammatory activity [7]. These data do not establish an immediate dose-dependent effect of alcohol on portal pressure, but support continued exposure as a clinically relevant disease driver beyond the fixed structural burden of cirrhosis. Compared with other etiologies, alcohol-related cirrhosis does not appear to follow a consistently distinct pattern of portal hypertension-related complications. In one contemporary cohort, survival and most cirrhosis-related events were similar across patients with decompensated ArLD, MetALD, and MASLD, although infection rates were lower in ArLD than in MASLD. Its clinical course is nevertheless modified by the possibility of persistent or recurrent alcohol exposure and by frequent malnutrition, frailty, extrahepatic comorbidity, and psychosocial vulnerability [8].

### 2.2. Severe Alcohol-Related Hepatitis

sAH is an acute inflammatory syndrome that typically follows sustained heavy alcohol consumption. It may occur without established cirrhosis; however, in the transplant setting it frequently presents in patients with advanced fibrosis or previously unrecognized cirrhosis [9,10,11]. The National Institute on Alcohol Abuse and Alcoholism (NIAAA) framework classifies alcohol-related hepatitis as definite, probable, or possible. Probable alcohol-related hepatitis is diagnosed clinically by recent onset or worsening of jaundice within 8 weeks; heavy alcohol use for at least 6 months, with less than 60 days of abstinence before jaundice; bilirubin greater than 3 mg/dL; aspartate aminotransferase greater than 50 but below 400 IU/L; an AST-to-ALT ratio greater than 1.5; and no major confounder. Definite disease requires histological confirmation, whereas possible disease includes atypical presentations or confounding factors. Histological confirmation should be considered when uncertainty could alter treatment or transplant decisions [12]. The severe phenotype, commonly defined by a Maddrey’s discriminant function of at least 32 and/or MELD greater than 20, carries high short-term risk [9,10]. Reported mortality is approximately 18.5% at 28 days and 37.7% at 6 months [13].

### 2.3. Acute-on-Chronic Liver Failure

ACLF is not universally defined. In this Perspective, we follow the EASL-CLIF/CANONIC system, which applies the CLIF-C Organ Failure score to liver, kidney, brain, coagulation, circulation, and respiration. ACLF is defined by acute decompensation of cirrhosis (ascites, bleeding, hepatic encephalopathy, bacterial infections), associated with one or more organ failures, and high short-term mortality (>15% at 28 days). Grade 1 comprises single kidney failure, or one non-kidney organ failure with renal dysfunction (creatinine 1.5–1.9 mg/dL) and/or grade I–II hepatic encephalopathy; grade 2 comprises two organ failures; and grade 3 comprises three or more [14,15]. This differs from the APASL framework, centered on an acute hepatic injury in patients with chronic liver disease (excluding steatosis), causing jaundice and coagulopathy followed within 4 weeks by ascites and/or encephalopathy, and from NACSELD-ACLF, which requires at least two extrahepatic failures among circulatory shock, grade III–IV encephalopathy, renal replacement therapy, and mechanical ventilation [16,17]. These systems identify different populations and should not be treated as interchangeable. In ArLD, ACLF may be precipitated by acute alcohol-related hepatic injury, including sAH, bacterial infection, or variceal bleeding [15,18]. Mortality increases with the number and severity of organ failures. For clinical purposes, two overlapping presentations can be distinguished: sAH presenting with ACLF, which is associated with poorer transplant-free survival and a greater burden of organ failures, and ACLF without sAH, in which ArLD is the underlying chronic liver disease but the acute episode may occur without evidence of histological alcohol-related hepatitis and be precipitated by bacterial infection, variceal bleeding, or another insult [19].

## 3. Non-Transplant Management and Dynamic Reassessment

In decompensated alcohol-related cirrhosis, achievement or maintenance of abstinence should accompany guideline-based management of ascites, portal-hypertensive bleeding, hepatic encephalopathy, infection, and kidney dysfunction, together with nutritional and frailty assessment [1,20].

In sAH, corticosteroids provide a short-term survival benefit in selected patients but are generally avoided in the presence of uncontrolled infection, active gastrointestinal bleeding, severe renal dysfunction, or multiorgan failure; treatment may be reconsidered after reversible contraindications have been controlled. Response should be assessed with the Lille score after 4–7 days and corticosteroids stopped in non-responders [9,13,21,22]. Concomitant ACLF, particularly at higher grades, further worsens prognosis and may reduce the likelihood of corticosteroid response [23]. No pharmacological rescue therapy has demonstrated a durable survival benefit. Anakinra plus zinc was inferior to prednisone in a randomized trial, and the phase 2b AHFIRM trial of larsucosterol did not meet its primary composite endpoint of 90-day mortality or LT [24,25].

Fecal microbiota transplantation (FMT) is an emerging non-transplant approach supported by the role of gut dysbiosis in ArLD. Pilot studies have explored FMT in steroid-ineligible sAH, AUD, and alcohol-related cirrhosis [26,27]. A 2026 meta-analysis suggested better survival than standard care, but studies were small and heterogeneous, certainty was low, and direct comparison with corticosteroids showed no significant benefit [28]. Its feasibility and safety in uncontrolled infection, gastrointestinal instability, or multiorgan failure remain uncertain; FMT should therefore remain within dedicated protocols and should not be regarded as an established rescue therapy or an alternative to LT.

In ACLF, non-transplant care depends on rapid treatment of precipitants and organ-specific support. Extracorporeal systems have been investigated as bridges to recovery or LT. In a randomized first-in-human study of 32 patients with alcohol-related ACLF, DIALIVE was safe and improved albumin function, inflammatory biomarkers, CLIF-C scores, and time to ACLF resolution, but did not reduce 28-day mortality and was not powered for survival [29]. Clinical response and organ-failure trajectory should be reassessed longitudinally while transplant evaluation proceeds in parallel.

### Delisting and Recompensation After Abstinence

Alcohol cessation is a disease-modifying intervention that may improve liver function and resolve decompensating events. According to Baveno VII, recompensation requires sustained removal or suppression of the primary etiological factor—sustained abstinence in ArLD—together with resolution of ascites without diuretics and hepatic encephalopathy without lactulose or rifaximin, and absence of recurrent variceal bleeding, maintained for at least 12 months, plus stable improvement in liver function [30,31]. Recompensation is a clinical state, not histological reversal of cirrhosis. When improvement is sufficiently durable to reduce the expected benefit of LT, it may lead to delisting; however, recompensation and delisting are related but not interchangeable outcomes.

Improvement-related delisting has been reported in approximately 2–16% of candidates with ArLD, depending on the population and transplant system [32]. US registry studies reported rates of 8.1–8.3% [33], while Pose and colleagues reported 8.6%; lower MELD and higher platelet count predicted improvement, and 76% of delisted patients remained free from liver-related death or LT at 5 years [34,35]. In a 17-center cohort of 633 patients with decompensated alcohol-related cirrhosis, 31.1% achieved abstinence-induced recompensation, with cumulative incidences of 12.3%, 23.4%, and 33.8% at 1, 2, and 5 years, respectively. Recompensation was more likely with early abstinence and in the absence of further decompensation and was associated with lower mortality [7].

Clinically significant portal hypertension and HCC risk may persist after recompensation; lifelong hepatology follow-up, semiannual HCC surveillance, and guideline-based reassessment of portal hypertension therefore remain necessary. Recurrent decompensation or worsening liver function should prompt renewed LT evaluation [30]. Continued AUD care is equally essential because patients remain at dual risk from residual cirrhosis and renewed alcohol-related injury; recurrent harmful alcohol use may reactivate the etiological driver and precipitate renewed hepatic deterioration [36]. Metabolic dysfunction may further limit recovery. In a single-center LT-evaluation cohort, recompensation was less frequent in MetALD than in ArLD alone (3% vs. 18%), while a 2026 multicenter cohort associated cardiometabolic risk factors, particularly diabetes and higher BMI, with lower recompensation and transplant-free survival [37,38]. These observational findings require external validation and should not determine candidacy or observation time in isolation.

## 4. Pre-Transplant Assessment

### 4.1. Evidence Supporting Early Liver Transplantation in Severe Alcohol-Related Hepatitis

The poor prognosis of medically nonresponsive sAH supports proceeding to LT in carefully selected patients without requiring a prolonged pre-transplant abstinence interval [21]. The pivotal 2011 study by Mathurin and colleagues showed substantially better 6-month survival after eLT than in matched non-transplanted controls [39]. Subsequent cohorts reproduced this benefit. ACCELERATE-AH reported survival of 94% at 1 year and 84% at 3 years among 147 recipients, and European experiences documented comparable outcomes [40,41]. In selected cohorts, post-LT survival is similar to that observed in patients transplanted for alcohol-related cirrhosis following a conventional pre-transplant abstinence period, and neither sAH itself nor shorter pre-LT abstinence has consistently predicted inferior survival [42]. Adoption nevertheless varies across countries because of organ availability, deceased- versus living-donor systems, cultural attitudes, and selection and allocation policies [43]. Living-donor LT may be particularly relevant where deceased-donor grafts are scarce, provided donor safety, voluntariness, and avoidance of coercion are rigorously protected [44]. The Dallas Consensus supports eLT in highly selected patients with sAH when medical nonresponse, absence of prohibitive comorbidity, and an acceptable multidisciplinary psychosocial profile are documented, without a fixed abstinence interval [42]. National policy remains heterogeneous [5]. German guidance, for example, generally requires 6 months of complete abstinence before wait-listing for alcohol-related cirrhosis, mental-health assessment, and serial alcohol-marker testing, but permits expert-reviewed exceptions in acute decompensated alcohol-related liver disease [45].

The favorable outcomes reported after eLT have limited external validity. The Mathurin cohort represented a small, stringently selected fraction of referred patients, while subsequent studies were mainly observational, used non-uniform selection and alcohol-use definitions, and remain vulnerable to referral, selection, ascertainment, and center effects. Systematic reviews are constrained by small samples, heterogeneity, and limited long-term comparisons [46]. In the QuickTrans trial, non-inferiority for alcohol-related outcomes was not demonstrated despite comparable survival [47]. Therefore, these findings should not be extrapolated to all medically nonresponsive patients, and eLT requires transparent selection and longitudinal addiction care.

### 4.2. Medical Assessment

Among selected recipients with ACLF, including grade 3, post-LT survival appears to be determined more by the severity and reversibility of organ failure than by the etiology of chronic liver disease. In a 15-center North American cohort, neither ArLD as the underlying etiology nor AH as the ACLF precipitant independently affected 1-year survival, although AH with ACLF was associated with greater post-LT healthcare resource use [48,49,50].

Patients with alcohol-related ACLF should therefore undergo the same time-sensitive medical assessment as those with ACLF of other etiologies. Evaluation should proceed alongside intensive care and treatment of precipitants and should serially assess organ-failure trajectory, infection control, hemodynamic and respiratory support, neurological status, frailty, and expected transplant benefit. What differs is the simultaneous need to assess AUD severity, treatment engagement, likely adherence, and the risk of harmful post-LT alcohol use. Recent alcohol use or the absence of sAH should not categorically preclude LT; a minority of critically ill patients may retain an acceptable profile after expedited multidisciplinary evaluation [4,51,52].

### 4.3. Psychosocial Assessment

A structured psychosocial assessment should be applied across all ArLD phenotypes, with its timing and depth adapted to clinical urgency. It should evaluate psychiatric and cognitive stability, co-occurring substance use, prior adherence, social support, and the feasibility of caregiving, housing, communication, and follow-up. Depression, anxiety, trauma-related disorders, and modifiable social or healthcare-access barriers should prompt treatment and mitigation rather than automatic exclusion. Addiction specialists, mental-health professionals, social workers, hepatologists, and transplant surgeons should contribute whenever feasible.

The Stanford Integrated Psychosocial Assessment for Transplantation (SIPAT) can structure the evaluation of readiness, support, psychiatric stability, lifestyle, and substance-use risk, but it is not alcohol-specific, and no universal cut-off has been validated across sAH, ACLF, and stable cirrhosis [53]. No single psychosocial characteristic or score should determine candidacy; structured instruments should complement clinical interviews, collateral information, and multidisciplinary judgment [4,42,51,54].

### 4.4. AUD Evaluation

AUD assessment is a distinct core component of transplant evaluation. It should characterize AUD severity and course, drinking trajectory, previous treatment and rehabilitation attempts, insight into alcohol-related harm, motivation, treatment engagement, co-occurring substance use, and the feasibility of longitudinal follow-up. Alcohol-related insight is clinically relevant but remains inconsistently defined and measured [54]. Direct alcohol biomarkers provide objective evidence of recent exposure and support longitudinal monitoring, but their interpretation requires integration with self-report, collateral information, and the broader clinical context [20,55]. Phosphatidylethanol (PEth) is measured in whole blood. Many programs use less than 20 ng/mL as compatible with no or low recent exposure and at least 200 ng/mL as suggesting repeated moderate-to-heavy use, but these cut-offs are not universal. PEth may remain detectable for weeks and should be interpreted with the assay, sampling interval, trend, and clinical history; interindividual kinetics, anemia or altered red-cell turnover, hemolysis, and recent transfusion can distort results, including transfusion-related false positives [56,57]. Because PEth is carried in erythrocyte membranes, transfused packed red cells can introduce donor-derived PEth and produce clinically relevant positivity for more than 2 weeks, particularly after multiple units [58]. In critically ill candidates, transfusion timing and dose, anemia, hemolysis, and altered red-cell turnover should therefore be reviewed before interpretation. Where feasible, sampling should precede transfusion; an unexpected post-transfusion positive result should prompt repeat testing after an appropriate interval and corroboration with a complementary biomarker rather than an immediate eligibility decision. Urine EtG generally reflects exposure over 1–3 days, although dose, hydration, renal dysfunction, bacterial degradation or formation, and incidental ethanol exposure affect interpretation. Hair EtG provides a months-long window: 5 pg/mg or less does not contradict abstinence, above 5 pg/mg supports repeated exposure, and at least 30 pg/mg supports chronic excessive consumption. Hair site and length, cosmetic treatment, growth variability, and renal dysfunction may cause misclassification [59]. Discordant results should prompt repeat testing and, when appropriate, interpretation alongside a complementary biomarker selected according to the relevant detection window; no biomarker should be used alone to deny listing.

### 4.5. AUD Treatment

AUD treatment should begin during LT evaluation or hospitalization and continue after transplantation. Core interventions include motivational interviewing, cognitive–behavioral therapy, strategies to prevent harmful return to alcohol use, peer support when acceptable, contingency management where available, treatment of psychiatric comorbidity, and mitigation of practical barriers. Embedding addiction clinicians within transplant programs can facilitate continuity, and observational integrated-care pathways have reported low rates of harmful post-LT alcohol use [60,61,62]. Small observational studies support the feasibility of tailored behavioral interventions, while a 2026 six-center cohort associated treatment participation with wait-listing but not with pre- or post-LT alcohol use and identified hospitalization and underinsurance as barriers to treatment access [63,64]. Prospective studies are needed to determine whether specific behavioral interventions improve alcohol-related and transplant outcomes. Pharmacotherapy should be selected jointly by hepatology, addiction, and pharmacy teams [61]. Baclofen has randomized-trial evidence in cirrhosis and limited hepatic metabolism, but sedation, dizziness, encephalopathy risk, and renal clearance require cautious titration [65]. Acamprosate has minimal hepatic metabolism and preliminary prospective data suggest acceptable tolerability in ArLD, although efficacy data remain limited; dose reduction is required when creatinine clearance is 30–50 mL/min, and the drug should be avoided at 30 mL/min or lower [66]. Naltrexone is effective in the general AUD population and may be considered in selected patients with stable liver function, but should be avoided in AH or severe hepatic decompensation and is contraindicated with current opioid use or anticipated perioperative opioid treatment. Gabapentin and topiramate are off-label options requiring renal dose adjustment and caution because of sedation, cognitive effects, and encephalopathy; disulfiram should be avoided because of hepatotoxicity. After LT, medication choice should be individualized according to graft and kidney function, neuropsychiatric effects, opioid requirements, and potential interactions with immunosuppression; evidence in transplant recipients remains limited [61]. Most first-line AUD medications are not major CYP3A4 inhibitors or inducers, but transplant-specific interaction data remain sparse. Practical risks are mainly indirect: calcineurin-inhibitor-associated kidney dysfunction can increase exposure to renally cleared acamprosate, baclofen, and gabapentin; baclofen, gabapentin, and topiramate may compound calcineurin-inhibitor neurotoxicity; and naltrexone blocks opioid analgesia. Topiramate is a mild CYP3A4 inducer, so tacrolimus, cyclosporine, or mTOR-inhibitor trough concentrations should be rechecked after initiation or dose escalation. Medication reconciliation, graft and kidney function, neurologic symptoms, and immunosuppressant troughs should be reviewed whenever AUD pharmacotherapy is started or titrated [61,67].

## 5. Outcomes After LT

### 5.1. Patterns of Post-LT Alcohol Use

LT for ArLD generally provides patient survival comparable with that observed for other transplant indications, including among carefully selected eLT recipients [40,42]. A historical meta-analysis restricted to recipients transplanted for ArLD included more than 8000 patients across 92 studies and estimated rates of any and heavy post-LT alcohol use of 22% and 14%, respectively, over a mean follow-up of 48.4 months [68]. However, definitions and ascertainment varied substantially, and most included studies predated contemporary eLT pathways and biomarker-based surveillance.

Comparative findings regarding eLT are inconsistent. QuickTrans and a contemporary single-center cohort reported more high-intensity or harmful alcohol use after eLT than after standard LT, whereas a PEth-monitored cohort found similar 1-year return-to-use rates across early and standard LT groups [47,69,70]. These findings do not establish eLT itself as an independent risk factor, because candidate selection, alcohol-use definitions, monitoring strategies, and follow-up differed across studies.

The clinical implications of post-LT alcohol use depend primarily on its timing, intensity, persistence, and consequences. Isolated or non-heavy use has not shown the same prognostic impact as sustained harmful use, which is associated with recurrent alcohol-related graft injury, progressive fibrosis, graft loss, and mortality [71]. The imprecise and potentially stigmatizing term “relapse” should therefore be avoided in favor of descriptions of clinically meaningful patterns and harm [42]. ACCELERATE-AH identified four trajectories after eLT for sAH: abstinent, late non-heavy, early non-heavy, and early heavy use. Five-year survival was 62% and 53% in the two early-use groups, compared with 95% and 100% in the abstinent and late non-heavy groups, respectively; younger age, multiple previous rehabilitation attempts, and pre-LT overt hepatic encephalopathy were associated with early-use trajectories [72].

Post-LT surveillance should combine non-stigmatizing interviews, collateral information when clinically appropriate, treatment engagement, and longitudinal interpretation of direct biomarkers. Harmful alcohol use should prompt intensified addiction treatment rather than disengagement from transplant care. In ACCELERATE-AH, adjusted predicted 5-year survival was 94% among recipients who achieved sustained reabstinence after harmful alcohol use, compared with 93% among those without harmful use and 77% among those with continued harmful use, supporting a treatment-oriented approach [73,74].

Alcohol-specific risk tools such as SALT and HRAR may complement, but not replace, multidisciplinary assessment. SALT was developed in eLT recipients with sAH, whereas HRAR reflects drinking history and prior treatment. Both have limited sensitivity and positive predictive value, although lower scores may help identify patients at low risk of harmful alcohol use [69,72]. Neither should determine candidacy in isolation.

### 5.2. Management of Post-LT Comorbidities

Long-term care should address both graft and non-graft risks. Cardiometabolic prevention includes management of blood pressure, lipids, diabetes, weight, nutrition, physical activity, smoking, and kidney function, with immunosuppression individualized to limit metabolic and renal toxicity. Cancer prevention should combine age- and sex-appropriate screening with skin surveillance and particular attention to tobacco- and alcohol-associated upper aerodigestive and lung malignancies. In recipients transplanted for HCC, post-LT surveillance should be individualized according to recurrence risk. Vaccination, infection-prevention counseling, and prompt evaluation of opportunistic or community infections remain essential. Adherence assessment should be routine because barriers to adherence are multifactorial and potentially modifiable [75]. Graft monitoring should include serial liver tests and immunosuppressant levels, with imaging or biopsy when indicated to investigate rejection, biliary or vascular complications, drug toxicity, and recurrent or de novo liver disease. These elements should be coordinated with AUD treatment and alcohol-use monitoring through collaboration among transplant hepatology and surgery, addiction medicine, psychiatry, pharmacy, primary care, and the patient’s support network [76,77].

## 6. Conclusions

LT for ArLD is moving from fixed abstinence thresholds toward dynamic, phenotype-informed care. Patients with decompensated cirrhosis require reassessment for recovery after alcohol cessation, whereas selected patients with medically nonresponsive sAH or ACLF may require expedited LT. Replacing a single time-based criterion with multidimensional evaluation of urgency, reversibility, transplantability, AUD severity, psychosocial vulnerability, and expected benefit does not lower selection standards but makes them more clinically relevant (Figure 1). The survival benefit of eLT in carefully selected patients is substantial, although its generalizability remains limited by stringent selection, heterogeneous protocols, and the concentration of experience in specialized programs. Long-term success requires integrated hepatology and addiction care, psychiatric and social support, biomarker-informed monitoring, and systematic graft and non-graft surveillance. Prospective studies across diverse clinical, cultural, and allocation settings are needed to validate selection pathways and determine which interventions most effectively prevent or treat harmful post-LT alcohol use.

## Figures and Tables

**Figure 1:**
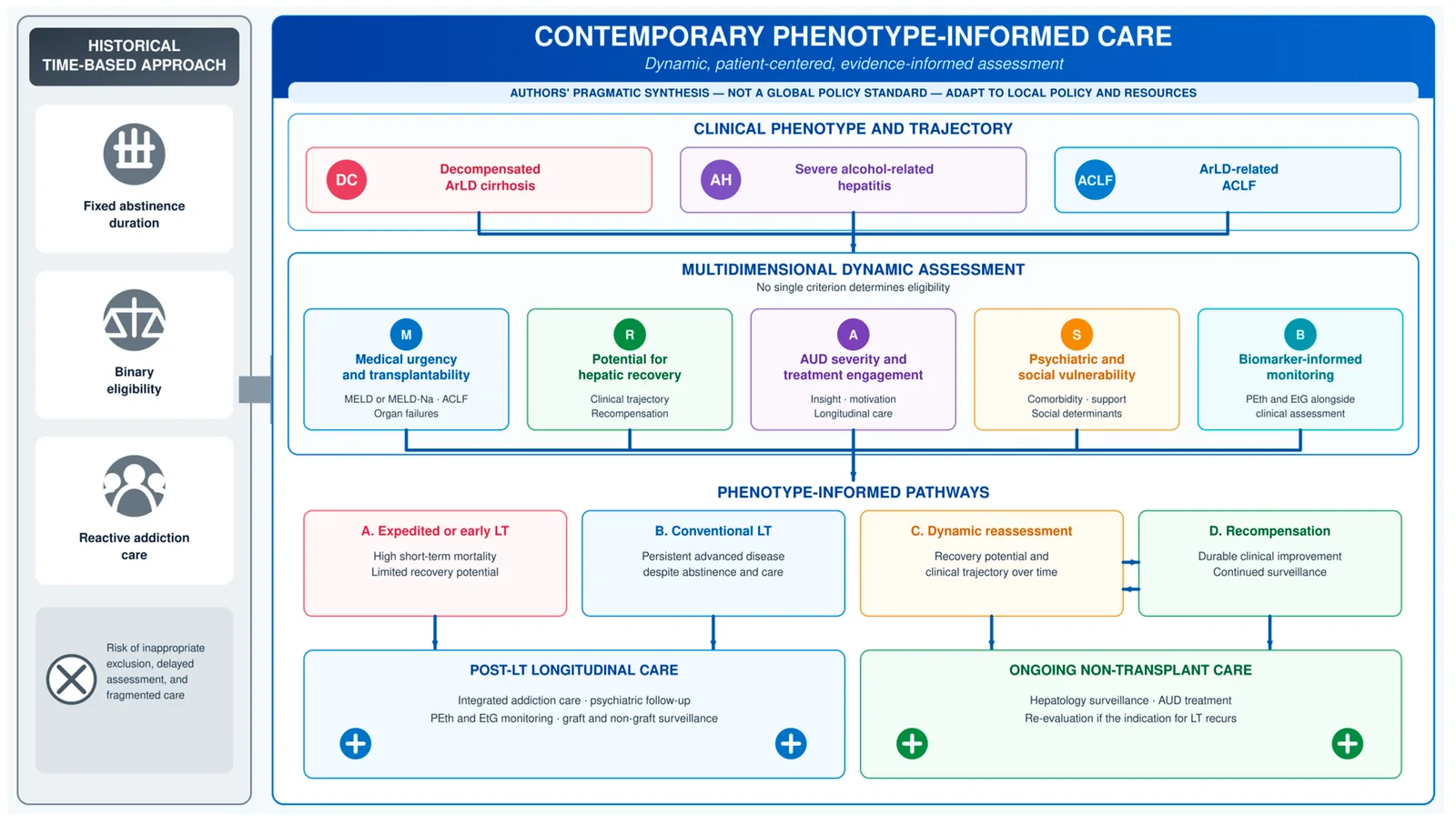
From a historical time-based approach to contemporary phenotype-informed liver transplant care in alcohol-related liver disease. The operational framework is the authors’ pragmatic synthesis rather than an established global policy standard and should be adapted to national regulation, allocation policy, organ availability, and local resources.

**Table 1:** Guideline-supported principles and authors’ proposed operational pathways for transplant-relevant phenotypes of alcohol-related liver disease. The authors’ proposed operational pathways represent a pragmatic synthesis rather than a formal international eligibility standard and should be adapted to national regulation, organ availability, and local resources. Abbreviations: ACLF, acute-on-chronic liver failure; ArLD, alcohol-related liver disease; AUD, alcohol use disorder; eLT, early liver transplantation; HCC, hepatocellular carcinoma; LT, liver transplantation; MELD, Model for End-Stage Liver Disease; MELD-Na, MELD-sodium; sAH, severe alcohol-related hepatitis.

Clinical Phenotype	Guideline- and Consensus-Supported Principles	Authors’ Proposed Operational Pathway
**Decompensated alcohol-related cirrhosis**	Use standard medical and multidisciplinary psychosocial evaluation. Abstinence duration should not be used as a stand-alone criterion for candidacy or as a predictor of harmful post-LT alcohol use. Treat AUD and assess recovery potential.	When clinically feasible, reassess over approximately 3–6 months using MELD/MELD-Na trajectory, decompensating events, and portal-hypertension burden. Proceed to conventional LT if recovery is insufficient; delist only after durable improvement or recompensation, with continued surveillance.
**Severe alcohol-related hepatitis**	eLT may be considered without a fixed 6-month abstinence interval in carefully selected patients with medical nonresponse or contraindications to corticosteroid therapy. Integrated addiction care is required throughout the LT continuum.	Confirm probable or definite sAH; consider transjugular biopsy if diagnostic uncertainty would alter treatment or LT decisions. Assess MELD, Lille response when applicable, organ failures, uncontrolled infection, prohibitive comorbidity, AUD severity, psychiatric stability, social support, and treatment engagement. Proceed to eLT only after multidisciplinary consensus.
**ArLD-related ACLF, with or without sAH**	Name the ACLF definition used. Urgently assess precipitants, reversibility of organ failures, operative risk, and futility. Recent alcohol use alone should not determine candidacy.	Use the EASL-CLIF grade and serial organ-failure trajectory. Require adequate infection control or response to treatment, potentially reversible organ failures, an acceptable perioperative-risk profile, and a feasible addiction/psychosocial plan. Proceed to eLT only while transplantability is preserved.
**Clinical improvement after abstinence**	Alcohol cessation is disease-modifying. Baveno VII recompensation requires etiological control and sustained resolution of decompensation; it is not histological reversal of cirrhosis.	Delist only after durable improvement and multidisciplinary review. Continue lifelong hepatology and AUD care, 6-month HCC surveillance, reassessment of portal hypertension, with variceal surveillance and prophylaxis when indicated; relist if the transplant indication recurs.

## Data Availability

No new data were created or analyzed in this study. Data sharing is not applicable to this article.

## Abbreviations

AASLD, American Association for the Study of Liver Diseases; ACLF, acute-on-chronic liver failure; ArLD, alcohol-related liver disease; AST, American Society of Transplantation; AUD, alcohol use disorder; eLT, early liver transplantation; EASL, European Association for the Study of the Liver; EtG, ethyl glucuronide; FMT, fecal microbiota transplantation; HCC, hepatocellular carcinoma; HRAR, High-Risk Alcoholism Relapse scale; HVPG, hepatic venous pressure gradient; ILTS, International Liver Transplantation Society; LT, liver transplantation; MELD, Model for End-Stage Liver Disease; MELD-Na, MELD-sodium; MetALD, metabolic dysfunction- and alcohol-related liver disease; NIAAA, National Institute on Alcohol Abuse and Alcoholism OF, organ failure; PEth, phosphatidylethanol; sAH, severe alcohol-related hepatitis; SALT, Sustained Alcohol Use Post-Liver Transplant; SIPAT, Stanford Integrated Psychosocial Assessment for Transplantation; UNOS, United Network for Organ Sharing.
